# Cognitive perspectives on maintaining physicians’ medical expertise: II. Acquiring, maintaining, and updating cognitive skills

**DOI:** 10.1186/s41235-023-00497-8

**Published:** 2023-07-25

**Authors:** Zachary A. Caddick, Scott H. Fraundorf, Benjamin M. Rottman, Timothy J. Nokes-Malach

**Affiliations:** 1 Learning Research and Development Center, University of Pittsburgh, 3420 Forbes Ave., Pittsburgh, PA 15260 USA; 2 Department of Psychology, University of Pittsburgh, Pittsburgh, PA USA

**Keywords:** Diagnosis, Expertise, Dual-process theory, Memory, Retrieval failure, Aging

## Abstract

Over the course of training, physicians develop significant knowledge and expertise. We review dual-process theory, the dominant theory in explaining medical decision making: physicians use both heuristics from accumulated experience (System 1) and logical deduction (System 2). We then discuss how the accumulation of System 1 clinical experience can have both positive effects (e.g., quick and accurate pattern recognition) and negative ones (e.g., gaps and biases in knowledge from physicians’ idiosyncratic clinical experience). These idiosyncrasies, biases, and knowledge gaps indicate a need for individuals to engage in appropriate training and study to keep these cognitive skills current lest they decline over time. Indeed, we review converging evidence that physicians further out from training tend to perform worse on tests of medical knowledge and provide poorer patient care. This may reflect a variety of factors, such as specialization of a physician’s practice, but is likely to stem at least in part from cognitive factors. Acquired knowledge or skills gained may not always be readily accessible to physicians for a number of reasons, including an absence of study, cognitive changes with age, and the presence of other similar knowledge or skills that compete in what is brought to mind. Lastly, we discuss the cognitive challenges of keeping up with standards of care that continuously evolve over time.

## Significance statement

Physicians’ expertise and ability to keep up with changing evidence is central for positive patient health outcomes. Here, we begin our review by first evaluating evidence of how expertise is acquired in the medical domain, considering dominant theories about the important benefits and detriments experience has in evaluating new information. We introduce complimentary evidence related to the role memory plays in learning and, since physicians often have careers that spans several decades, the impact that aging and time since initial certification may have on physicians' clinical performance. Importantly, medicine is an ever-evolving field which leads to changes to the standards of care over time. In light of this reality, we discuss how evidence from the cognitive science literature can inform how best to address the challenge of this complex information environment. Given the wide scope of this endeavor, we conclude with proposals to fill in important gaps in knowledge that may continue to propel the field of medicine and physician assessment forward to ensure the highest standards of care possible.

## Introduction

A primary goal of continuing certification programs is to ensure that board-certified physicians maintain at least a certain minimum level of expertise. In this article, we examine what principles and findings from cognitive science imply about acquiring, maintaining, and updating medical expertise. We first discuss psychological theories of the acquisition of higher-order cognitive medical skills, diagnosis in particular. Next, we discuss how learned skills can be maintained in the face of forgetting and age-related changes in cognition. Lastly, we discuss the process of updating and acquiring new knowledge as medical practice evolves.

We focus on medical decision making and expertise from the traditional information-processing perspective of cognition that undergirds cognitive psychology. We acknowledge that medical decision making is much more complex in that it occurs situated in a dynamic environment with other physicians and healthcare professionals and in the larger context of medical systems (see the 2020 special issue of the journal *Diagnosis*, Volume 7, Issue 3, for many articles on this perspective). However, because continuing certification program assessments only test a physician’s cognitive abilities independently, not their performance in a clinical environment, we focus on the individual physician’s cognitive skills.

To situate the strength of the evidence and claims made, we attach evidence levels (EL) to in-text citations for empirical claims (see Table 1). Evidence levels range from 1 to 6, with 1 being the strongest evidence (meta-analyses) and 6 being the weakest (opinion papers).

**Table 1:** Evidence levels for in-text citations for empirical claims

Evidence level	Type of work
1	Quantitative meta-analysis
2	Narrative review
3	Multiple original experiments/randomized controlled trials (RCTs)
4	Single original experiment/RCT
5	Correlational or quasi-experimental study
6	Opinion paper

This article is part of a collection of five articles in this special issue focused on how physicians maintain medical expertise across their careers. These reviews are narrative reviews, not systematic, because they cover a wide variety of topics, not a single narrow topic.

## Acquiring medical expertise

Expertise is marked by the acquisition of large amounts of knowledge, which in turn affects how information is organized, represented, and processed. General aptitude measures struggle to predict expert performance, suggesting that expertise is not just reserved for the highly intelligent (Moneta-Koehler et al., 2017, EL: 5). Rather, experiences play an important role in the development of expertise. For instance, the amount of *deliberate practice*—activities designed to improve targeted aspects of performance—that an individual has completed predicts their level of expertise (Ericsson et al., 1993, EL: 5). It is likely the *quality* of practice, rather than quantity, that is necessary to develop expertise; the mere number of deliberate practice hours on their own does not adequately explain expert performance (Macnamara et al., 2014, EL: 1). Ample and accurate feedback is also crucial (Kahneman & Klein, 2009, EL: 2). In the process of developing expertise, individuals learn to categorize information based on abstract principles, whereas novices categorize based on superficial details (Chi et al., 1981, EL: 3).

### Dual-process theories in medical decision making

A common theme in the cognitive psychology literature is the existence of two distinct systems for information processing (for overviews, see Evans, 2008; Kahneman & Frederick, 2002; Sloman, 1996). (The term “systems” in this literature refers to cognitive strategies and habits, not necessarily to neural or anatomical distinctions.) Most dual-processing theories hold that System 1 is fast, unconscious, evolutionarily old, associative, and universal. In contrast, System 2 is slow, conscious, evolutionarily new, and rule based. An important difference between the systems is that System 2 is under the control and guidance of the individual whereas System 1 occurs automatically. Although it may seem intuitive that the conscious and controlled System 2 is superior to System 1, this is not always the case. System 1, at times, produces highly accurate decisions and does so quickly and from little information (Marewski & Gigerenzer, 2012).

Within medicine, the dual-process theory is widely accepted as the dominant paradigm for understanding clinical decision making generally, and especially for diagnosis (Croskerry, 2009a, EL: 2; 2009b, EL: 2; Croskerry et al., 2013a, 2013b, EL: 2; Norman & Eva, 2010, EL: 2; Pelaccia et al., 2011, EL: 2). For example, the dual-process theory provides the theoretical backbone of the Institute of Medicine’s report on Improving Diagnosis in Healthcare (National Academies of Sciences, Engineering, & Medicine, 2015, Chapter 2, EL: 2).

System 2 is understood as the analytical, hypothetico-deductive reasoning or problem-solving process. For example, a physician might diagnose a patient by systematically running one test, ruling out one diagnosis, and then following it up with a different test relevant to a different potential diagnosis—a process that involves a series of carefully thought-out decisions with logical reasoning. This sort of logical reasoning was studied extensively in the earlier years of research on medical decision making (e.g., Elstein et al., 1978; Barrows et al., 1982; Coderre et al., 2003; Newfeld et al., 1981). However, one of the broad conclusions of this research is that changes in hypothetico-deductive problem solving do not appear to explain the transition from novices to experts (e.g., Boshuizen & Schmidt, 1992; Elstein & Schwarz, 2002; Groen & Patel, 1985; Neufeld et al., 1981; Norman, 2005). Instead, it seems that experts use a variety of different forms of knowledge and representations. This is not to say that hypothetico-deductive problem solving is not used by experts; of course, someone with insufficient medical training cannot effectively engage in this sort of reasoning, and of course experts do slow down, ask for second opinions, consult resources, and engage in other forms of careful analytical thinking. Rather, the point is that experts have additional skills and knowledge, one of which is extensive experience with individual patients.

In contrast to System 2, System 1 is the non-analytic decision making in medicine is the very fast pattern recognition process. For instance, pattern recognition allows a physician to quickly think of hyperthyroidism when seeing a patient who is skinny, tremulous, perspiring a lot, and has bulging eyes and a swollen neck. Pattern recognition involves classifying a current patient as similar to a prior patient (called an *exemplar*) or similar to an abstracted pattern of multiple prior patients with the same disease (called a *prototype*). Though most often discussed in terms of diagnosis, this pattern recognition process is also relevant to other decisions, such as deciding whether to order further diagnostic testing, choosing a treatment, or deciding whether to refer to a specialist. Pattern recognition is believed to rely on the same cognitive processes that people use every day, e.g., for categorizing animals as dogs versus cats or for identifying different species of trees (Cohen & Lefebvre, 2017).

Another aspect of non-analytic System 1 decision making in medicine is the use of *heuristics*—mental shortcuts that allow decisions to be reached quickly and efficiently. Pattern recognition through categorization can in fact be viewed as one such heuristic (e.g., Nilsson et al., 2008), though heuristics are broader than just pattern recognition (e.g., National Academies of Sciences, Engineering, & Medicine, 2015, Chapter 2; Whelehan et al., 2020). For example, the *representativeness* heuristic leads physicians to judge the probability that a patient has a given disease based on the *sensitivity* of the diagnostic information (probability of a positive test given that the patient has the disease) rather than its *positive predictive value* (probability that a patient has a disease given a positive test; Casscells et al., 1978; Eddy, 1982; Rottman, 2017). This is appropriate when the two diseases are roughly equally prevalent, but leads to *base rate neglect* when one is more common.

Heuristics are neither inherently bad nor inherently good. They can help physicians make fast decisions, which can be critical in situations with time pressure. And, simple rule-based heuristics sometimes outperform formal statistical regression analysis (Marewski & Gigerenzer, 2012, EL: 2). On the other hand, sometimes heuristics are applied in the wrong context or are overly simple, such as in the base rate neglect example, which can lead to suboptimal decisions.

Despite the fact that the dual-process theory is widely accepted as the dominant model of clinical decision making, there are important debates, open questions, and ambiguities with this model. Some researchers question whether there is a clear distinction between the two systems and whether there are only two systems (e.g., De Neys, 2021, 2022; Evans, 2008). Other research has challenged the assumption that they are always opposed to each other and instead may work together (Cushman & Morris, 2015; see Kool et al., 2018 for a review). Though the two systems are often presented as always coming to different decisions, it is likely that they would often come to the same decision in a given situation (De Neys, 2022), which raises a question of how to distinguish which of the two systems is responsible for a given decision.

Perhaps the most pressing question is how people coordinate between the two systems. For example, does some sort of signal of low confidence in System 1 lead to the engagement of System 2, or are both systems engaged simultaneously with a discrepancy-monitoring system noticing when they are coming to different decisions (De Neys, 2022)? These are not just important questions for psychology but are directly related to medicine. Moulton et al. (2007) proposed that being an expert in medical decision making involves “slowing down when you should.” This emphasis on “when you should” raises the questions of when an individual should slow down and how this slowing down works. Moulton et al. (see also Croskerry, 2009b) argue that these questions are exactly what the field needs to address to truly understand medical expertise and how to support and train expertise. Stated another way, instead of focusing on fast and slow thinking processes individually, it is more important to understand how experts know that they need to slow down and think a bit harder in certain situations; unfortunately this question is hard to address and there is little existing research.

### The role of experience in medical decision making

Here we take a slightly different approach from the dual-process model. This review focuses on the role that experience (interactions with many patients over time) plays in expertise. Experience with prior patients is at the core of non-analytic System 1: recognizing the pattern in the current case as similar to prior cases. In contrast, relying on rules, evidence, guidelines, and knowledge of pathophysiological and pharmacology rather than one’s own idiosyncratic past experience fits with System 2. We do not claim that prior experiences with individual patients cannot be part of the analytical model of decision making and slow deliberative thought. Indeed, of course it is possible that when making a decision about a current patient that a physician may carefully and analytically make comparisons to individual prior patients; in naturalistic decision making it is not possible to know whether a physician is engaged in ‘slow’ or ‘fast’ thinking at a given moment (or that only one process occurred). However, to the extent that extensive experience can produce very fast decisions, it is more likely to be involved in System 1 thinking.

The strength of utilizing experience is that it allows for fast pattern recognition, which frequently leads to accurate diagnoses. For example, one study (Norman et al., 1989, EL: 5) examined the accuracy of dermatologists reading slides. They found that not only did the dermatologists demonstrate high levels of accuracy, but they also answered significantly faster on slides they got correct, showing how diagnosis can often be both extremely fast and accurate. A number of studies with primary care and emergency medicine physicians have found similar results: clinicians think of a few potential diagnoses within seconds to minutes and are usually right (Barrows et al., 1982, EL: 5; Elstein et al., 1978, EL: 5; Gruppen et al., 1988, EL: 5; Pelaccia et al., 2014, EL: 5). This has led to the provocative question by Norman et al. (2007): “How can it be that experts with minimal information are able to advance tentative hypotheses about the diagnoses, seemingly effortlessly, and apparently without conscious awareness of the retrieval process? … Where do the hypotheses come from?” The answer, according to this line of research, is that with enough experience clinicians can quickly pattern-match a target case from a large set of prior cases.

However, there are also downsides to relying heavily on experience. Even though pattern recognition and reliance on the diagnosis and treatment decisions made for past patients can often work out well, it can also lead to biases. For example, in one experiment, family medicine residents were given a set of cases to practice interpreting ECGs. In the initial set of cases, a brief clinical scenario accompanied each case, along with the correct diagnoses. In the latter set of test cases, participants had to identify the correct diagnosis. For some of the test cases, the accompanying clinical scenario involved irrelevant features, such as the patient’s job, that matched features from the initial cases. When an irrelevant feature matched a prior case, the residents were more likely to give the same diagnosis as the prior case, which turned out to be wrong (Hatala et al., 1999, EL: 4; for other similar studies, see Brooks et al., 1991, EL: 4; Young et al., 2011, EL: 4).

A few studies that shown a similar role of prior experience in real-world medical decision making. One study investigated how often physicians prescribed warfarin for patients with atrial fibrillation in order to prevent a stroke, which despite the risks, is a standard of practice (Choudhry et al., 2006, EL: 5). When one of the physician’s patients who was on warfarin experienced a severe bleeding event that was likely a side effect of the warfarin, the physicians were about 20% less likely to prescribe warfarin for patients with atrial fibrillation for at least a year afterward. Another study found that after delivering a baby and experiencing labor and delivery complications, a physician was a bit more likely to use a vaginal delivery instead of cesarean, or vice versa, depending on the prior delivery method (Singh, 2021). In sum, physicians’ decisions can be impacted by recent experiences with other patients.

A particular challenge is that these experiences are idiosyncratic. If a physician works in a specialized clinic, they may see certain types of patients even though they still need to be able to diagnose and treat a broader set of patients that they see less frequently. This means that physicians are systematically missing out on experience with certain types of patients. For example, in one study, residents’ beliefs about the prevalence of a disease were correlated with their probability of providing it as a potential diagnosis (Rottman et al., 2016, EL: 5). In general, this tendency makes sense from the rational Bayesian perspective of diagnosis, in which general “prior” beliefs about the likelihood of diseases in the population are updated with knowledge of the signs and symptoms and diagnostic tests of the specific patient to form a “posterior” probability of each disease on the differential (Ledley & Lusted, 1959; Pauker & Kassirer, 1980). However, to the extent that prevalence beliefs are distorted by one’s experience, some diagnoses could be overlooked. Additionally, the appearance of patients with rare diseases or rare side effects from treatments (e.g., the bleeding events discussed above) is governed by chance, so physicians may be influenced by the vicissitudes of daily practice. Thus, it is important to receive corrective feedback and not to overly rely on one’s own experiences.

Another problem with relying on one’s experience is that experience provides an imperfect feedback system. Feedback is vital for developing expertise (e.g., Ericsson, 2015, EL: 2; Hattie & Timperley, 2007, EL: 2; Kahneman & Klein, 2009, EL: 2), and lack of feedback is believed to contribute to overconfidence (Kahneman & Klein, 2009, EL: 2). However, the medical system is poor at providing feedback (National Academies of Sciences, Engineering, & Medicine, 2015, EL: 6; Schiff, 2008, EL: 6). An error in diagnosis or treatment may never be discovered, in which case feedback is never received. Additionally, because of the complex nature of modern medicine and the fact that an individual patient often has contact with many physicians, an individual physician often never knows the outcomes of patients that they encountered, resulting in a lack of both negative and positive feedback. For these reasons, two Institute of Medicine reports (McGinnis et al., 2013; National Academies of Sciences, Engineering, & Medicine, 2015; see also Rosner et al., 2023) have called for healthcare organizations to create better feedback systems.

In summary, accumulating experience with many patients is believed to be a critical aspect of how doctors become experts. However, due to working in specialized practices, chance encounters with certain patients but not others, and imperfect feedback systems, doctors will not always experience (and re-experience) certain types of patients. For this reason, one value of a longitudinal continuing certification program is to provide physicians with a well-rounded set of vignettes with feedback to supplement their real-world experiences. We recommend prioritizing gaps that are likely to occur in a physician’s practice as well as those that have a meaningful impact on the quality of care delivered.

## Maintaining expertise

### Cognitive skills decline over time

**Fig. 1:**
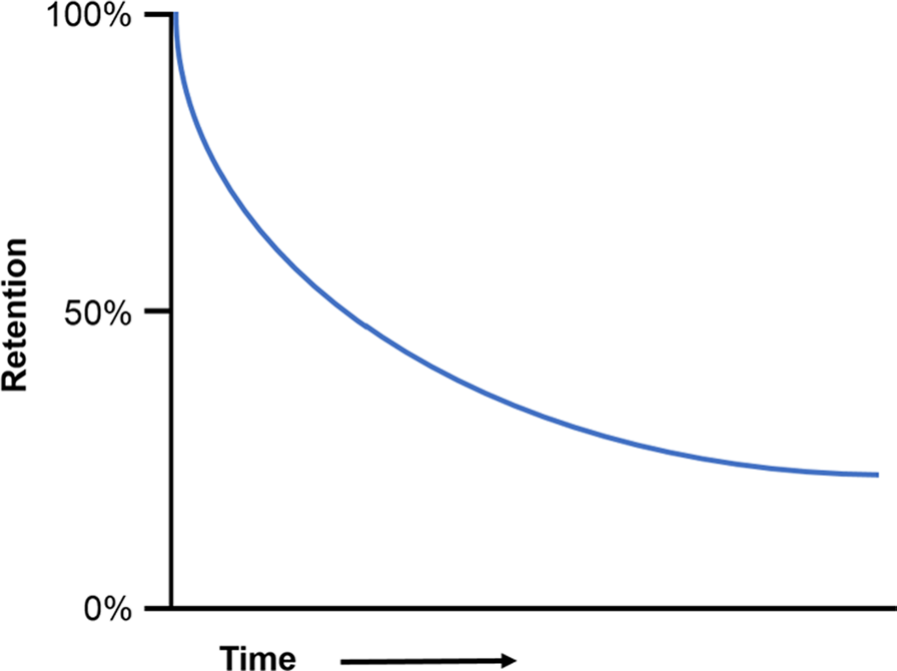
Prototypical forgetting curve

Once learned, cognitive skills must be maintained: in the absence of study, learned information and procedures are forgotten over time. Decades of research suggest that, across content domains and types of tasks, forgetting tends to follow a negatively accelerated *power law* function such that a great deal of material is forgotten initially, but the remaining material is forgotten more slowly (Ebbinghaus, 1885, EL: 4; Rubin & Wenzel, 1996, EL: 2; Wickelgren, 1974, EL: 4; Wickens, 1998, EL: 6; Wixted, 2004, EL: 3; Wixted & Carpenter, 2007, EL: 3). That is, people forget much of what they see and hear in the initial minutes and hours afterward, somewhat more in the following days and weeks, and comparatively little of what remains in the months and years ahead (see Fig. 1). This power-law function may reflect the rate at which people are likely to stop re-encountering those topics (Anderson & Schooler, 1991, EL: 5).

In terms of medical expertise, the power law suggests that some knowledge will be retained relatively well over long periods of time, but it is almost inevitable that other material will be quickly forgotten after being encountered in training if it is not deliberately practiced. How often important information needs to be practiced is likely to vary across individuals and be influenced by other variables. Nevertheless, the rapid decline in retention after any given study episode suggests that it is beneficial to distribute study over time to alleviate these losses.

### Reasons for forgetting

Why do people forget? One intuitive hypothesis might be that we simply run out of mental “storage space” and that prior knowledge is forced out to make room for the new. And, it is indeed clear that there are sharp restrictions on how much can be held in *working memory*, or what we are currently thinking about (although the specification and cause of those limits remain debated; e.g., Cowan, 2010, EL: 2; Miller, 1956, EL: 2). However, it is not clear that there are practical limits on the total capacity of long-term learning and knowledge (Drachman, 2005; Landauer, 1986). Laboratory studies have demonstrated that people can readily learn and remember hundreds or even thousands of pictures or sentences even after only seconds of exposure to each (e.g., Shepard, 1967, EL: 4; Standing, 1973, EL: 4; Standing et al., 1970, EL: 4). Indeed, the cortex of the human brain contains approximately 150 trillion (1.5 × 10^14^) synapses (Drachman, 2005, EL: 3). That is orders of magnitude more than what the average individual knows: 40,000 (4 × 10^4^) words (Brysbaert et al., 2016, EL: 3), 750 (7.5 × 10^2^) people (Zheng et al., 2006, EL: 4), or, more generally, approximately 1 billion (10^9^) bits of information (Landauer, 1986, EL: 2).

Another intuitive hypothesis as to why we forget is that skills and knowledge are lost to *decay*; that is, memories fade and are simply lost over time. This hypothesis receives more support; given the regularities in how memories decline over time (discussed above), it is likely that the passage of time contributes to forgetting (Wixted, 2004: EL 2; Sadeh et al., 2014: EL 2). However, decay is not likely to be the *whole* story, since not all memories are fated to be lost over time: People can remember the names and locations of buildings on their college campus (Bahrick, 1983, EL: 5) or the names and faces of their high school classmates (Bahrick et al., 1975, EL: 5) even after decades of disuse.

Thus, cognitive psychologists often emphasize *interference* from other, similar information as an additional cause of forgetting. Many things commonly forgotten in daily life are those that compete with many other similar memories. For example, it is often difficult to remember where I left my phone this morning because I have many other competing memories of other places where I left my phone at different times. One experimental demonstration of interference is the *fan effect* (Anderson, 1974, EL: 3; Anderson & Reder, 1999, EL: 4): Learning multiple overlapping associations makes any individual association harder to retrieve (e.g., learning *the lawyer is in the cave* and *the lawyer is on the beach* is harder than learning *the lawyer is in the cave* and *the fireman is on the beach*). Thus, categorizing individual exemplars is more difficult (slower) for broader categories (e.g., “cancer” or “plants”) than for more narrow ones (e.g., “red-green colorblindness” or “flowers”; Landauer & Freedman, 1968, EL: 3; Landauer & Meyer, 1972, EL: 2; c.f., Collins & Quillian, 1970, EL: 3). Interference can happen both *proactively*, when old knowledge makes it harder to learn competing new knowledge (Watkins & Watkins, 1975, EL: 2), and *retroactively*, when new knowledge, once acquired, interferes with retrieving old knowledge (Postman & Underwood, 1973, EL: 2).

Indeed, interference-based failures to retrieve *some* information may be an inevitable consequence of remembering *other*, competing information (*retrieval-induced forgetting*; Anderson et al., 1994, EL: 2; Roediger, 1978, EL: 2). Imagine the process of diagnosing a patient with chest pain. Retrieving *myocardial infarction* in response to the cue *chest pain* reinforces thinking of *myocardial infarction* for future cases, and it also correspondingly weakens the likelihood of considering *aortic dissection* as a diagnosis. Thus, less common concepts and information are particularly vulnerable to interference (Anderson et al., 1994, EL: 3). This suggests it should be particularly important for physicians to practice similar and easily confusable concepts, especially those similar to more common concepts (e.g., common diagnoses) and thereby vulnerable to retrieval-induced forgetting.

### It is not always adaptive to retrieve

The phenomenon of retrieval-induced forgetting relates to another key property of human memory: At least in some cases, it is beneficial or adaptive *not* to bring all of one’s knowledge to mind (Bjork, 1989, EL: 2; Kuhl et al., 2007, EL: 4; MacLeod, 1998: EL 2; Nørby, 2005, EL: 2; Popov et al., 2019, EL: 3; Wimber et al., 2015, EL: 4). In the case of retrieval-induced forgetting, for instance, it is likely beneficial on the whole to prioritize frequently used facts and concepts over those less frequently used, so as to reduce interference and cognitive demands (Bäuml & Samenieh, 2010, EL 4; Kuhl et al., 2007, EL: 4; Nørby, 2005, EL: 2; Popov et al., 2019, EL: 3; Wimber et al., 2015; EL: 4). For instance, standards of care change (as we discuss below), and it could be beneficial for physicians not to bring to mind outdated standards. Indeed, when explicitly told that some information is obsolete or otherwise should now be forgotten, people can prioritize study and retention of other, to-be-remembered information (for more discussion of the mechanisms of such *directed forgetting*, see MacLeod, 1998; EL: 2; Sahakyan et al., 2013, EL: 2). Forgetting the details of individual episodes or exemplars (e.g., individual patients) can also facilitate learning broader patterns or prototypes (e.g., diagnoses), supporting System 1 pattern recognition system, as discussed above (Nørby, 2005, EL: 2; Posner & Keele, 1968; EL: 3).

Thus, it is unlikely that it would be possible or even desirable to eliminate forgetting completely. Another implication is that is not necessarily advisable for physicians to try to remember every detail of every case, and they are perhaps better served by abstracting more general principles. Lastly, it may be valuable to explicitly highlight when standards of care or other information is out of date so that physicians can leverage directed forgetting to prioritize current, relevant knowledge and skills.

### Inaccessible knowledge can often be recovered or relearned

Although people may sometimes be unable to bring to mind the desired knowledge or skills, that does not necessarily mean the learning is lost forever. Knowledge that is forgotten at one point in time can sometimes spontaneously be retrieved later (a phenomenon known as *hypermnesia*; Erdelyi & Becker, 1974, EL: 3). A classic example is the *tip-of-the-tongue* phenomenon (e.g., Burke et al., 1991, EL: 5), when one has a sense of knowing a particular word or name but being unable to retrieve it, only to spontaneously recover it later. Thus, failure to retrieve an idea at any point in time is not necessarily, or even likely, diagnostic of permanent loss. The fact that inaccessible knowledge and skills are not fully lost also leads to *savings* in that previously encountered knowledge can be relearned more quickly than it was initially acquired (Ebbinghaus, 1885, EL: 4; Nelson, 1978, EL: 4).

Although inaccessible knowledge can sometimes be retrieved spontaneously, it is more apt to be retrieved with appropriate *retrieval cues* (e.g., Tullis & Benjamin, 2015, EL: 2; Tullis & Fraundorf, 2017, EL: 3), characteristics of the environment that helps to “jog” one’s memory (although certain cues can be unhelpful if they disrupt a planned retrieval strategy; Basden & Basden, 1995, EL: 3; Roediger, 1978, EL: 3). In general, human memory is partially context-dependent, such that memories more readily come to mind when the environment relates to them or matches how they were initially learned or acquired (Bjork & Richardson-Klavehn, 1989, EL: 2). More frequent longitudinal assessment, rather than point-in-time assessment, could thus serve as a cue to keep this knowledge accessible and/or facilitate relearning.

## Effects of age on memory and learning

Beyond the time that has elapsed since medical training, another source of skill decline may be aging. We first cover the basic science of aging and then address studies of aging specifically as it relates to physicians.

### Age affects some cognitive skills more than others

A clear conclusion from the basic science of memory aging is that age differentially affects different types of knowledge. Beginning in early adulthood (e.g., age 20), performance steadily declines with age on tasks that require *fluid intelligence*; that is, those that involve novel learning or reasoning (Horn & Cattell, 1966, EL: 5; Horn & Cattell, 1967, EL: 5). This decline may be driven at least in part by declines in more fundamental aspects of cognition: The speed of even very basic cognitive processing (e.g., as measured by the speed of identifying whether two strings of letters are the same or different) declines with age, as does the ability to temporarily hold information in *working memory* (Park et al. 2002, EL: 5; Salthouse, 1991, EL: 5; Salthouse, 1996, EL: 2; Salthouse, 2004, EL: 2; Salthouse, 2005, EL: 5; Salthouse & Babcock, 1991, EL: 5; Stine-Morrow et al., 2008, EL: 5). For instance, declines in basic speed may drive age differences in more complex tasks insofar as cognitive skills may break down if people cannot retrieve or compute relevant information sufficiently quickly to be useful for the task at hand (Hertzog et al., 2003, EL: 5; Salthouse, 1991, EL: 5; Salthouse, 1996, EL: 2; Salthouse & Babcock, 1991, EL: 5; Salthouse, 2005, EL: 5; Stine-Morrow et al., 2008, EL: 5). Declines in processing speed are relevant to many areas of medicine because physicians often see high volumes of patients in a day, need to address multiple problems per visit, and in some settings need to switch quickly between patients. It is not enough simply to have acquired the relevant cognitive skills; physicians need to be able to bring to mind—or know where to look up—the relevant knowledge in time to be practically useful.

By contrast, fixed knowledge, often referred to as *crystallized intelligence*, is preserved or even increases with age (Horn & Cattell, 1966, EL: 5; Horn & Cattell, 1967, EL: 5; Park et al., 2002, EL: 5; Salthouse, 2004, EL: 2; Zacks & Hasher, 2006, EL: 2). Even fixed knowledge may decline at especially advanced ages (e.g., age 80 or above; Park et al., 2002, EL: 5; Salthouse, 2004, EL: 2), but physicians would likely be retired at this age. In general, then, older adults rely less on novel (fluid) episodic learning and more on existing (crystallized) knowledge about the world (Castel, 2005, EL: 4; Castel, 2007, EL: 4; Castel et al., 2013, EL: 3; Koutstaal & Schacter, 1997, EL: 3; McGillivray & Castel, 2017, EL: 3; Stine-Morrow et al., 2008, EL: 4; Zacks & Hasher, 2006, EL: 3). This has mixed implications for the retention and use of medical expertise: On the one hand, physicians’ general medical knowledge might be expected to be relatively spared with age. On the other hand, older physicians might be less proficient at learning new techniques or remembering newly encountered cases and patients.

Further, although older adults underperform younger adults even in very basic memory tasks, age differences are larger in some types of learning and retrieval than others (Fraundorf et al., 2019, EL: 1). For instance, it has been argued that older adults are especially challenged by cognitive skills that require self-initiated or controlled processing, such as deliberately committing novel information to memory (e.g., learning new standards of care) or systematically reviewing one’s memory (e.g., deliberately considering each of a series of potential diagnoses). By comparison, age is less deleterious for relatively automatic or habitual uses of memory, such as applying a familiar set of actions (e.g., ordering a frequent diagnostic test) or recognizing a stimulus (e.g., recognizing a familiar set of symptoms as a particular disease) (e.g., Craik, 1986, EL: 2; Hoyer & Verhaeghen, 2005; EL: 2; Luo & Craik, 2008, EL: 2; c.f., Fraundorf et al., 2019, EL: 1). This pattern is consistent with age-related declines in the controlled, analytical System 2 but preserved or enhanced functioning of the automatic, experience-based System 1 (Eva, 2002, EL: 2; Eva, 2003, EL: 2). It suggests that older physicians may rely heavily on habitual, rather than new, cognitive skills and that they will better remember patients and treatments consistent with their experience.

Several other generalizations regarding memory aging highlight other situations where older physicians’ cognitive skills might be preserved. First, older adults perform comparatively well at remembering new information that is naturalistic (as opposed to arbitrary laboratory stimuli; Castel, 2007, EL: 4) or that allows the use of existing everyday memory strategies, such as establishing routines or leaving reminders for oneself (Bailey et al., 2010, EL: 4; Moscovitch, 1982, EL: 5; Rendell & Craik, 2000, EL: 3; Rendell & Thomson, 1999, EL: 3). Second, older adults are *as* or even *more* effective than younger adults at working with familiar partners to remember information as a team. These *collaborative cognition* strategies can include dividing responsibilities for remembering different kinds of information and suggesting cues to support each other’s memory (Dixon & Gould, 1996, EL: 3; Dixon, 1999, EL: 2). Third, older adults are sensitive to indicators of the *value* of to-be-retained information and perform comparatively well in remembering material that is important or that otherwise aligns with their motivational priorities. For instance, in laboratory experiments, older adults are adept at prioritizing material that prioritizing material that is worth more “points” toward a goal (Castel, 2007, EL: 3; Castel et al., 2002, EL: 3; Castel et al., 2007, EL: 3), that a speaker emphasizes intentionally (Fraundorf et al., 2012, EL: 4), that aligns with a motivational bias for positivity (Charles et al., 2003, EL: 3; Mather & Carstensen, 2005, EL: 3; May et al., 2005, EL: 3), or that comes from a more trustworthy source (Rahhal et al., 2002, EL: 3). Indeed, even non-physician older adults better remember fictive medications with severe side effects than those with less severe side effects (Hargis & Castel, 2018, EL: 3). All three of these age-related changes would be expected to favor retention of medical skill and learning even with increasing age insofar as physicians use their medical expertise in everyday life, often work with well-established teams, and (presumably) value their medical knowledge and skills.

However, a final generalization is that there is clear meta-analytic evidence that older adults are especially impaired in remembering the *source* or *context* of information (Fraundorf et al., 2019, EL: 1; Old & Naveh-Benjamin, 2008, EL: 1; Spencer & Raz, 1995, EL: 1). This could have deleterious consequences in medicine if older physicians confuse or misattribute the symptoms or treatments prescribed to several patients they have recently seen.

In sum, there is reason to be optimistic that physicians can retain much of their general medical knowledge with increasing age. However, older physicians may be vulnerable to reduced memory for specific cases or patients, and they may access their knowledge more slowly.

### Aging as it relates to physicians

The role of aging in physicians’ cognitive skills has been addressed in a number of narrative reviews (e.g., Ajmi & Aase, 2021, EL: 2; Eva, 2002, 2003; Durning et al., 2010; Williams, 2006; EL: 2, Council on Medical Education, 2015, EL: 2). Assessing the role of age in a physician’s ability to provide high-quality care is quite complicated because a number of factors are so highly correlated that it is usually impossible to distinguish them.

First, it is possible that a physician’s abilities decline with age due to memory or processing decline. Second, it is possible that knowledge and skills could decline due to the passage of time out of medical school and residency; this variable is highly confounded with age among physicians insofar as most physicians enter medical school at roughly similar ages. Third, it is possible that as the number of years since residency increases, a physician’s knowledge becomes out of date due to shifting standards that they did not learn in medical school or residency. Fourth, over time a physician accumulates more direct patient experience, which as explained above can have both positive and potentially negative impacts on performance. Fifth, some physicians specialize over time, which could lead them to lose broader skills that have become less relevant to their practice. In the following paragraphs, we unpack evidence relevant to aging physicians. When we mention correlations with age, we acknowledge that many other factors, explained above, are highly correlated with age. Thus, we are using “age” as a proxy variable, and this is not meant to implicate cognitive aging as the reason for these associations.

Reliable evidence indicates that the quality of healthcare provided decreases with physician age. A systematic review of 62 studies found that 45 studies (73%) reported a decrease in performance for some or all outcomes (Choudhry et al., 2005, EL: 2). Another 13 (21%) found no association. The remaining four (6%) found a non-linear (inverted U) trend or an increase in some or all outcomes. This pattern held across a wide variety of measures, including knowledge measures, health outcomes, and adherence to standards of care for diagnosis, screening, prevention, and therapy. However, one potential limitation of this review is that it covered a period of time during which evidence-based medicine and quality-assurance techniques, such as performance evaluation, were becoming adopted. So, it is possible that the apparent age-related declines may instead be driven by the fact that the older physicians were trained prior to this shift and that the newer generation of physicians, who were trained to value evidence-based medicine, may not exhibit declines in quality of care as they age if they stay up to date with the evidence.

Since this systematic review, several notable studies reinforce this pattern of decreases in quality of care provided by older physicians. A population-based study of adherence to guidelines for antibiotic prescribing in treating urinary tract infections in children in Taiwan found that adherence dropped gradually from 87% in physicians younger than 35 to 45% in physicians older than 55 (Chen et al., 2011, EL: 5). Holmboe et al., (2008, EL: 5) found that physicians more than 20 years out of medical school performed considerably worse on the maintenance of certification exam compared to physicians fewer than 20 years out. Physicians who scored lower on the assessment also exhibited worse performance on measures of treating patients: whether they had diabetes patients obtain eye exams, lipid tests, and HbA1c tests, whether they had female patients receive a mammogram in the past year, and whether they had patients with coronary artery disease obtain a lipid test in the past year. St-Onge et al. (2015, EL: 5) similarly found worse diagnostic performance for clinical vignettes among older physicians. In sum, there is evidence from multiple sources of a general decrease in both conceptual knowledge and quality of care with increased age.

However, the specific reasons for the decreasing quality of care with age is less certain. One possibility already discussed (see also Eva, 2002, EL: 2), is that performance decline may be caused by negative changes in cognitive processing. Another possibility is that older physicians fail to learn and/or retain changing standards of care. In fact, another study of scores on the ABIM test found that age predicted poorer performance on questions that tested knowledge for standards of care that had changed over the preceding 30 years, but age did not predict poorer performance on questions about standards of care that had not changed (Day et al., 1988, EL: 5; see also Holmboe et al., 2008). However, this study is quite dated, and it is not certain that this finding would still hold among the current cohort of physicians, who participate in different forms of continuing education than physicians 30 years ago. More studies of this nature could help elucidate why knowledge and performance appear to decline with age.

Although performance generally declines with age, there are some cases where it may not. System 1 (non-analytical processing or pattern recognition) may remain stable or even improve with age and experience; in the previous section, we discussed how more automatic forms of memory or habitual responses, as well as fixed knowledge, remain intact until advanced ages of 80  or above (see also Eva, 2002, EL: 2). Being able to continue to rely on automatic forms of memory accords with the important role of non-analytical medical decision making. Some studies have indeed found that older physicians tend to both identify correct diagnoses very quickly and settle on a diagnosis quickly. This is a double-edged sword. On the one hand, it can lead to quick and accurate diagnoses. For example, two studies (Eva et al., 2010, EL: 5; Hobus & Schmidt 1993, EL: 5; see discussion in Eva, 2002) found a positive relation between age/years of experience and diagnostic accuracy. However, quickly settling on a diagnosis can also lead to premature closure (i.e., failing to consider alternatives after reaching a decision), and some research has found that older physicians focus more heavily on information presented earlier in a case (Eva & Cunningham, 2006, EL: 5).

In sum, the majority of the evidence suggests that older physicians perform worse in a variety of ways, even though gaining experience over one’s career may mitigate this decline to some extent. However, doctors are tasked not only with maintaining current standards of care but also keeping up with changing standards, which we discuss in the next section.

## Keeping up with changing standards of care

One of the fundamental challenges in medicine is keeping up with the ever-changing standards of care. An Institute of Medicine report (McGinnis et al., 2013) concluded that diagnostic and treatment options are changing at an accelerating rate, making it ever more important to keep up with changing standards. Two major reviews have systematized the barriers to using current standards of care (Cabana et al., 1999, EL: 2; Cochrane et al., 2007, EL: 2). Though the reviews differ in many ways, there is substantial agreement in terms of the cognitive and attitudinal barriers identified. Imagine a physician learning about a new treatment standard of care. First, the physician must become *familiar* with and *aware* of this new standard of care. Second, the physician must develop knowledge or skill, for example, knowledge about indications and dosages of a therapy. Third, the physician must form a high *outcome expectancy* (believe the treatment or standard would be beneficial) and *agree* with the new standard of care. Fourth, the physician should feel confident they can implement it, termed *self-efficacy*. By contrast, a physician may feel (for example) uncomfortable providing treatment for a condition at the boundary of their scope of practice. Fifth, the physician must overcome *habits* or *inertia*, doing things the same way as they have always been done.

How might a longitudinal assessment program affect these barriers? In Fraundorf et al. (2022), we discuss the overwhelming evidence that repeated testing benefits learning and retention and protects against interference from previously learned practices. Thus, longitudinal assessment would likely improve familiarity, awareness, and knowledge. It is less clear whether longitudinal assessment could impact the attitudinal barriers, such as outcome expectancy and self-efficacy, though it is possible that providing feedback (correct answers, explanations, and citations) might alleviate attitudinal barriers. Aside from longitudinal assessment, continuing medical education (CME) is the primary system currently in place designed to help physicians maintain cognitive skills and gain new skills. In the first article in this collection (Rottman et al., 2023), we discuss the strength and limitations of CME and compare CME to longitudinal assessment.

## Proposed studies and future directions

### Providing feedback about strengths and weaknesses and tracking age gaps over time

Two benefits of a longitudinal assessment program are that it can potentially help physicians learn about standards of care that have changed since their training and that it can provide useful feedback to physicians about their relative strengths and weaknesses. We propose that Boards prospectively classify items as testing new standards of care versus testing old–but still relevant–standards of care. This classification can then be used in three ways.

First, physicians can be provided with feedback about performance on these two different types of questions. This will provide physicians with a sense of whether they are challenged more by staying current versus by maintaining older knowledge.

Second, we propose that boards use an approach pioneered in a clever study (Day et al., 1988). This study looked at older versus younger physicians’ performance on the continuing certification program assessment, contrasting questions for which standards of care have changed over time versus questions for which they have not. The finding was that older physicians performed worse for questions testing knowledge about standards that had changed, but not for standards that had remained the same. Ideally, if the assessment program works to keep physicians up to date, this interaction for older vs. younger physicians on changed vs. unchanged standards should diminish over time. This analysis could be conducted both before and after implementing the longitudinal assessment program to evaluate the contributions of the educational component of the longitudinal program. And, it could be conducted on an ongoing basis to measure the success of the program over time, with the goal of continuously optimizing the test to minimize age differences.

Third, extending this analysis pioneered by Day et al. (1988) can help to uncover the reasons for poor performance. In particular, Day et al. found decreases in performance over time only on questions for which standards of care have changed over time, which seems to implicate challenges of staying current rather than aging or time since residency per se. However, since 1988, the landscape of CME has changed considerably, so it is not clear whether the same pattern would be found. Furthermore, one possibility is that physicians selectively keep up with standards that they think are especially relevant to their practice. This possibility could be assessed by having physicians rate the relevance of each question, and testing whether relevance interacts with age and whether or not a standard has changed. Still other analyses would be possible if physicians also rate their confidence in their answers. For example, one possibility is that if a physician is wrong on a question that involves a new standard, but is highly confident, that might mean old knowledge is interfering with learning new knowledge or that they never learned the new standards. In contrast, if a physician is wrong but not very confident on a question involving a new standard, that might indicate that a new standard has not been learned.

### Measuring response time during testing

Some prior research has examined the relationships between age, response time, accuracy, and case difficulty (Barrows et al., 1982, EL: 5; Elstein et al., 1978, EL:5; Gruppen et al., 1988, EL: 5; Norman et al., 1989, EL: 5; Pelaccia, et al., 2014, EL: 5). However, these findings are nuanced and not entirely consistent, and this research could benefit from broader case materials and from larger, more representative samples of physicians across many specialties. In fact, given that many items on continuing certification program assessments already incorporate questions about diagnosis and treatment, and response time is easy to record in a computer system, data could be easily obtained to test these relationships.

### Identifying out-of-date information

As we discussed above, laboratory evidence indicates that learners have the capability to engage in directed forgetting of material that has been explicitly cued as to-be-forgotten (e.g., because it is out of date or incorrect). There may be opportunities to leverage this capacity in longitudinal assessment; for example, by presenting outdated standards of care and explicitly indicating they are no longer current and should not be retained. We hypothesize that this should lead to better retention of current standards of care than a procedure in which out-of-date material is not explicitly addressed.

### Debiasing and clinical reasoning interventions

A more open-ended suggestion is to consider using longitudinal assessment programs as a platform to test debiasing and clinical reasoning interventions. In particular, the dual-process theory presumes that there are two systems of reasoning, one that is faster and one that is slower, and that these systems need to be coordinated (Croskerry, 2009a, EL: 2; 2009b, EL: 2; Norman & Eva, 2010, EL: 2; Pelaccia et al., 2011, EL: 2). For example, one proposal is that physicians need to learn how to switch from faster automatic judgment for routine problems to slower, more effortful reasoning for more unusual or ill-defined problems (Moulton et al., 2007; see also Graber, 2009). Croskerry and colleagues have reviewed potential ways to attempt to teach physicians to avoid common biases (e.g., Croskerry et al., 2013a; b).

Though several debiasing and clinical reasoning interventions have been proposed and tested, there is only mixed evidence whether they work (e.g., Schmidt & Mamede, 2015, EL: 2; see also Isler et al., 2020, for research on debiasing training outside medicine). Still, a number of consenses still express interest in such interventions (Olson et al., 2019; National Academies of Sciences, Engineering, & Medicine, 2015, pp. 4–32 to 4–34; Parodis et al., 2021), and new debiasing interventions are being tested with some promise (Kuhn et al., 2020; Mamede et al., 2020). Longitudinal assessment programs could serve as a testing ground for brief interventions that could be embedded right before or during a question.

In summary, there are a number of potential ways that data collected from longitudinal assessment programs, or from interventions embedded inside the programs, could be used to assess the efficacy of the programs themselves, provide guidance about how to improve feedback, and test basic questions about medical expertise and diagnosis that are hard to study in other settings and for which studies are rare and small. Many of these proposals can be accomplished with minimal changes to the duration of the program, and physicians may find them insightful if the results can demonstrate strong evidence regarding the roles of aging and keeping up with standards of care, speed versus accuracy, and debiasing techniques.

### Examining basic mechanisms of non-analytical reasoning in diagnosis

Longitudinal assessment also provides opportunities to further uncovering the basic mechanisms of non-analytical reasoning in medical decision making. Experiments on the role of non-analytical reasoning in diagnosis can be directly embedded into a longitudinal assessment program and, by doing so, would help to firmly establish an empirical base of knowledge regarding non-analytical reasoning in medicine. The research on non-analytical reasoning in medicine has only been tested in a few studies with only modest sample sizes (Brooks et al., 1991, EL: 4; Hatala et al., 1999, EL: 4; Young et al., 2011, EL: 4). Though these findings are intuitive, and though they build upon an extensive literature on the basic science of categorization from cognitive science (Cohen & Lefebvre, 2017), there exist important gaps in knowledge. First, most of the basic science research has been conducted with abstract stimuli, and with undergraduates who are trained for only short periods of time, not complex real-world medical stimuli that require years for physicians to master. Thus, conducting more and larger studies with physicians will help to establish these phenomena within medicine with more certainty.

A longitudinal assessment also provides a remarkable opportunity to design studies embedded into the training programs to test the role of irrelevant information from prior cases in biasing the diagnosis of future cases. This research could test how long the bias lasts, how strong the bias is, how the prevalence of a disease or a physician’s knowledge of a disease affects the bias, and whether age of the physician interacts with non-analytical reasoning. Further, given that most of the literature on non-analytical reasoning in diagnosis has focused on diagnosis based on visual information (e.g., reading ECGs, pathology, dermatology, radiology), another question is whether the bias is different for diagnosis that requires integrating multiple signs, symptoms, and lab reports (emergency medicine, internal medicine, etc.; Norman et al., 2007).

In sum, although there is broad consensus that non-analytic reasoning plays some role in diagnosis, we know comparatively little about *when*, *where*, and *how much* it matters. Delineating how experience both bolsters and biases subsequent decisions could help make physicians more aware of how such non-analytic factors could impact decision making, which could increase motivation to follow evidence-based guidelines or be used in debiasing efforts.

### The effect of feedback on outcome expectancy and self-efficacy

Another opportunity is to study the feedback provided that explains whether an answer is right or wrong. Among the multiple barriers to keeping up with changing standards of care, the main barriers that a longitudinal assessment program is intended to address are awareness, familiarity, and knowledge about the new standard of care. However, it is possible that feedback could also address other barriers, such as not believing that the new standard is better (outcome expectancy) and not being confident in how to implement it (self-efficacy). Experiments could be designed that manipulate the provided feedback, and questions could be embedded about a physician’s feelings of outcome expectancy and self-efficacy in order to test ways to maximize the broader utility of the feedback for overcoming multiple barriers.

## Summary and conclusion

We discussed four topics related to how physicians acquire, maintain, and update cognitive skills. First, we reviewed the dual-process theory of medical expertise, which proposes that medical decision making is a combination of (a) fast intuitive thinking (non-analytical processing) shaped by experience and (b) slow analytical thinking guided by logic. According to this theory, the benefits of non-analytical thinking are that decisions can be reached very fast and are often correct. However, intuitive, non-analytical thinking also has downsides. Idiosyncratic experiences can shape a physician’s decisions (e.g., about treatment decisions). And, idiosyncrasies in which patients a physician does and does not see affect the maintenance of expertise; for instance, they could distort the physician’s beliefs about the prevalence of a diagnosis and the likelihood of that diagnosis coming to mind.

Second, we discussed the basic science of why people forget or fail to retrieve information. Forgetting can sometimes be an adaptive in that it allows people to discard information that is out of date or less important, and forgetting individual details can help people to learn broader patterns. Nevertheless, it is clear that people sometimes fail to bring relevant information to mind. But, this does not necessarily indicate it is permanently lost. Rather, it may become accessible again later, especially with the right cues. One important cause of retrieval failures is the inability to access knowledge quickly enough to be useful. Another is interference from competing concepts and skills. The literatures both on interference and on analytical thinking point toward an opportunity for a longitudinal continuing certification program to attempt to fill in potential gaps in experience by testing cases that are somewhat rare but of high importance to patient care.

Third, whereas crystallized intelligence (e.g., medical knowledge) remains intact until age 80, fluid intelligence (e.g., novel learning or using balancing multiple tasks in working memory) declines with age. The majority of the evidence suggests that the quality of healthcare declines with physician age. This could be driven in part by the decline in fluid intelligence; however, another contributor could be a failure to keep up with changing standards of care.

Fourth, we reviewed physician-level barriers to keeping up with changing standards of care, including not being aware of or knowledgeable about a new standard, not believing that the new standard is better, not being confident in how to implement the new standard, and habits. The goal of continuing certification programs has traditionally been to assess whether physicians are keeping up with changing standards. The switch to a longitudinal assessment program presents the opportunity of serving both as assessment and as an educational program to make physicians more knowledgeable about new standards and their application in patient care.

## Data Availability

Not applicable.
